# Mean diffusivity associated with trait emotional intelligence

**DOI:** 10.1093/scan/nsz059

**Published:** 2019-10-08

**Authors:** Hikaru Takeuchi, Yasuyuki Taki, Rui Nouchi, Ryoichi Yokoyama, Yuka Kotozaki, Seishu Nakagawa, Atsushi Sekiguchi, Kunio Iizuka, Yuki Yamamoto, Sugiko Hanawa, Tsuyoshi Araki, Carlos Makoto Miyauchi, Kohei Sakaki, Yuko Sassa, Takayuki Nozawa, Shigeyuki Ikeda, Susumu Yokota, Magistro Daniele, Ryuta Kawashima

**Affiliations:** 1 Division of Developmental Cognitive Neuroscience, Institute of Development, Aging and Cancer, Tohoku University, Sendai 980-8575, Japan; 2 Division of Medical Neuroimaging Analysis, Department of Community Medical Supports, Tohoku Medical Megabank Organization, Tohoku University, Sendai 980-8575, Japan; 3 Department of Radiology and Nuclear Medicine, Institute of Development, Aging and Cancer, Tohoku University, Sendai 980-8575, Japan; 4 Creative Interdisciplinary Research Division, Frontier Research Institute for Interdisciplinary Science, Tohoku University, Sendai 980-8575, Japan; 5 Human and Social Response Research Division, International Research Institute of Disaster Science, Tohoku University, Sendai 980-8575, Japan; 6 Advanced Brain Science, Institute of Development, Aging and Cancer, Tohoku University, Sendai 980-8575, Japan; 7 School of Medicine, Kobe University, Kobe 650-0017, Japan; 8 Division of Clinical Research, Medical-Industry Translational Research Center, School of Medicine, Fukushima Medical University, Fukushima 960-1925, Japan; 9 Department of Human Brain Science, Institute of Development, Aging and Cancer, Tohoku University, Sendai 980-8575, Japan; 10 Division of Psychiatry, Tohoku Medical and Pharmaceutical University, Sendai 983-8536, Japan; 11 Department of Behavioral Medicine, National Institute of Mental Health, National Center of Neurology and Psychiatry, Tokyo 187-8553, Japan; 12 Department of Psychiatry, Tohoku University Graduate School of Medicine, Sendai 980-8575, Japan; 13 Advantage Risk Management Co., Ltd, Tokyo 153-0051, Japan; 14 Department of Language Sciences, Graduate School of Humanities, Tokyo Metropolitan University, Tokyo 192-0397, Japan; 15 Research Center for the Earth Inclusive Sensing Empathizing with Silent Voices, Tokyo Institute of Technology, Tokyo 152-8550, Japan; 16 Department of Ubiquitous Sensing, Institute of Development, Aging and Cancer, Tohoku University, Sendai 980-8575, Japan; 17 Department of Sport Science, School of Science and Technology, Nottingham Trent University, Nottingham, UK, NG11 8NS

**Keywords:** emotional intelligence, mean diffusivity, diffusion tensor imaging, dopaminergic system, somatic marker circuitry, social cognition

## Abstract

Previous neuroimaging studies have suggested that the neural bases of trait emotional intelligence (TEI) lie in the social cognition network (SCN) and the somatic marker circuitry (SMC). The current study was the first to investigate the associations of total TEI factors and subfactors with mean diffusivity (MD) of these networks as well as regional MD of the dopaminergic system (MDDS). We found that TEI intrapersonal factor score and total TEI score were negatively correlated with regional MDDS in the vicinity of the right putamen and right pallidum and that TEI intrapersonal factor score was negatively correlated with MD values of the fusiform gyrus. Total TEI score and TEI factor scores were positively correlated with MD values of various areas within or adjacent to SCN components, SMC structures and the lateral prefrontal cortex (LPFC). Our MD findings demonstrated the importance of the dopaminergic system to TEI and implicate the SCN, SMC and LPFC in TEI. Future studies are required to investigate the implications of positive and negative associations with MD values.

## Introduction

It has been shown that emotional intelligence (EI) can predict performance in various situations, particularly social and emotional situations (Van Rooy and Viswesvaran, 2004; Ciarocchi *et al.,* 2005). Furthermore, EI has distinctive characteristics compared with other psychometric constructs such as intelligence and temperament (Van Rooy and Viswesvaran, 2004; Ciarocchi *et al.,* 2005). It is generally accepted that there are two types of EI: trait emotional intelligence (TEI), which is typically measured by self-report questionnaires, and ability EI (AEI), which is typically measured by performance in certain cognitive tests. Both constructs have strengths and weakness for predicting other psychometric variables as well as common and distinct characteristics, which are summarized in our previous study (Takeuchi *et al.,* 2013a). There are several conceptualizations of EI. Among them, the Bar-On model (1997b, 2000) describes TEI as ‘a cross-section of interrelated emotional and social competencies, skills and facilitators that impact intelligent behavior, measured by self-report’ (Bar-On, 1997). And our recent studies have focused on TEI rather than AEI based on findings of intact AEI in Asperger syndrome (Montgomery *et al.,* 2008; Montgomery *et al.,* 2010). This is because autistic traits, which cause impaired abilities in social interactions, are important for the neuroscience as well as concepts of EI.

Previous neuroimaging studies have implicated three major neural circuits in TEI. The first is the social cognition network (SCN), which is involved in a number of impaired cognitive processes, such as theory of mind and recognition of biological motion, in persons with autism. In the current study, the SCN is defined to include regions such as the medial prefrontal cortex (mPFC), precuneus and superior temporal sulcus associated with social interactions, an important component of EI. The second is the somatic marker circuitry (SMC), which was defined in the somatic marker hypothesis (Damasio *et al.,* 1991). This hypothesis posits that emotion-based biasing signals arise from the body and that higher-order brain regions, such as the ventromedial prefrontal cortex (VMPFC), integrate these signals and in turn influence complex decision making (Damasio *et al.,* 1991). This construct overlaps substantially with the definition of EI as described above. The SMC includes regions such as the VMPFC, anterior insula, anterior cingulate cortex (ACC), somatosensory cortices, amygdala, basal ganglia and brainstem areas. The third network is the dopaminergic system, which partly overlaps with the SMC. As previously summarized (Takeuchi *et al.,* 2015c), greater EI is associated with greater self-motivation (Extremera and Fernández-Berrocal, 2005), and the ability to motivate oneself is a central concept of EI (Goleman, 1998; Uchiyama *et al.,* 2001). Moreover, numerous studies have revealed that the dopaminergic system is a major regulator of motivation (Takeuchi *et al.,* 2015c).

Consistent with the importance of these networks for TEI, previous neuroimaging studies have revealed that TEI is associated with neural mechanisms of SCN and SMC. These previous neuroimaging studies have used regional gray matter density (Takeuchi *et al.,* 2011), regional gray matter volume (Killgore *et al.,* 2012), white matter structure (Takeuchi *et al.,* 2013b), lesions (Bar-On *et al.,* 2003), brain activity during the perception of fearful faces and a visually based social judgment task (Killgore and Yurgelun-Todd, 2007; Smith *et al.,* 2017) and resting-state functional connectivity (Takeuchi *et al.,* 2013a). In addition, we found that a polymorphism of the dopamine receptor D2 is associated with TEI, which in turn is associated with motivational state in females (Takeuchi *et al.,* 2015c). In contrast, previous neuroimaging studies have failed to show associations between the dopaminergic system and TEI. However, to our knowledge, such studies have not investigated the associations between microstructural properties of the dopaminergic system and TEI.

Mean diffusivity (MD) in diffusion tensor imaging (DTI) reflects the diffusivity of water and a greater tissue density, such as the presence of more cellular structures, which prevents the free diffusion of water molecules and lowers the MD value (Assaf and Pasternak, 2008; Ni *et al.,* 2010; Sagi *et al.,* 2012). As we reviewed previously (Takeuchi and Kawashima, in press), MD in areas of the dopaminergic system (MDDS), particularly in subcortical areas such as the putamen, caudate and globus pallidum, are associated with various dopaminergic system functions. Evidence for the importance of MDDS in the dopaminergic system function can be summarized as follows:

(i) MD values in some areas of the dopaminergic system such as the caudate and putamen significantly and negatively correlated with dopamine synthesis capacity as measured by positron emission tomography (PET) (partial correlation coefficient ≈ 0.7) (Kawaguchi *et al.,* 2014).
(ii) MDDS is more sensitive in detecting Parkinson’s disease (a major dopaminergic system pathology) than other magnetic resonance imaging (MRI) measures and dopamine-related PET measures (Seppi *et al.,* 2004; Péran *et al.,* 2010).
(iii) MDDS is robustly and consistently associated with states and traits that in turn have been associated with dopaminergic function, such as novelty seeking, motivational state, fatigue and extraversion (Takeuchi and Kawashima, in press).
(iv) MDDS can also detect plasticity caused by environmental factors and interventions that are known to alter the dopaminergic system function (Razek *et al.,* 2011; Sagi *et al.,* 2012; Takeuchi *et al.,* 2014b).

The purpose of this study was to investigate the associations between regional MD values, particularly MDDS, and TEI. As in our previous studies (Takeuchi *et al.,* 2011; Takeuchi *et al.,* 2013b), we assessed TEI using the Japanese version of the Emotional Intelligence Scale (Uchiyama *et al.,* 2001). This scale consists of three factors: intrapersonal, interpersonal and situation management.

The intrapersonal factor (comprising questions related to self-insight, self-motivation and self-control) evaluates (i) self-awareness, (ii) the ability to act appropriately and (iii) the ability to control one’s behavior. The interpersonal factor (comprising questions related to empathy, altruism and interpersonal control) evaluates the ability to maintain adequate personal relationships based on empathy and understanding of another person’s emotions. The situation management factor (comprising questions related to insight into a situation, leadership and control over a situation) evaluates an individual’s ability to (i) endure and adapt to change, (ii) provide leadership and (iii) be flexible in the control and use of their abilities in dynamic situations (Uchiyama *et al.,* 2001).

We hypothesized that MDDS and MD of SCN and MD of SMC are associated with TEI or its factors (general hypothesis). Specifically, we hypothesized that MDDS is primarily associated with the intrapersonal factor because this factor includes components related to self-motivation and motivational components are associated with MDDS (Takeuchi and Kawashima, in press) (specific hypothesis 1). We also hypothesized that the MD of the key nodes of SCN such as the mPFC, posterior medial cortex and superior temporal sulcus is associated with the intrapersonal and interpersonal factors (specific hypothesis 2). This is because previous studies have shown that the intrapersonal and interpersonal factors are associated with other neural measures of these areas (Takeuchi *et al.,* 2011, 2013b). Finally, among the important nodes of SMC, we hypothesized that the MD of the anterior insula is associated with MD of the intrapersonal factor and the MD of the orbitofrontal cortex is associated with the situation management factor (specific hypothesis 3). This is because such associations have been observed for neural measures other than MD (Takeuchi *et al.,* 2011). Considering the focus on these multiple networks and the fact that the nature of MD is still being explored, we used the standard whole-brain approaches instead of region of interest approaches.

## Material and methods

### Subjects

The present study, which is a part of an ongoing project to investigate the associations among brain imaging measures, cognitive functions and aging, included data from 1207 healthy, right-handed individuals (693 males and 514 females). The following descriptions are mostly reproduced from one of our previous studies within the same ongoing project using the exact same methods regarding these issues (Takeuchi *et al.,* 2015a). The mean age of the subjects was 20.8 years [standard deviation (SD), 1.8]. All subjects were university students or postgraduates with normal vision and no history of neurological or psychiatric illness. Handedness was evaluated using the Edinburgh Handedness Inventory (Oldfield, 1971). Written informed consent was obtained from all adult subjects. For nonadult subjects, written informed consent was obtained from parents or guardians. All study procedures were approved by the Ethics Committee of Tohoku University. For more details, see Supplemental Methods.

### Emotional intelligence scale

The Japanese version of the Emotional Intelligence Scale (Fukunishi *et al.,* 2001; Uchiyama *et al.,* 2001) was used to assess TEI as in our previous studies (Takeuchi *et al.,* 2011; Takeuchi *et al.,* 2013b). The following descriptions were reproduced mainly from our previous studies using the exact same methods regarding these issues (Takeuchi *et al.,* 2011; Takeuchi *et al.,* 2013b). The Emotional Intelligence Scale is a self-report measurement that provides an estimate of EI and social intelligence. The scale was developed and standardized for use with Japanese subjects. Detailed descriptions of the development and psychometric properties of this questionnaire are included in the Emotional Intelligence Scale technical manual (Uchiyama *et al.,* 2001). The Emotional Intelligence Scale comprises 65 items scored on a 5-point Likert scale from ‘not true of me’ to ‘very often true of me’. Subjects’ responses were categorized into the following three composite scale scores or factors: (i) intrapersonal, (ii) interpersonal and (iii) situation management. Each composite scale score is composed of three subscale scores.

For more information of validity and reliability of this scale, see Supplemental Methods.

### Psychometric measures of general intelligence

The Raven’s Advanced Progressive Matrix (RAPM) (Raven, 1998) is a relatively pure measure of fluid reasoning to adjust the effects of general intelligence in the analyses. For more details, please refer to the Supplemental Methods for the procedures and rationales of this measure.

### Image acquisition

The methods for MR image acquisition were described in our previous study (Takeuchi *et al.,* 2016b). Briefly, all MRI data were acquired using the same 3T Philips Achieva scanner across 7 years. Diffusion-weighted data were acquired using a spin-echo EPI sequence [TR = 10 293 ms, TE = 55 ms, big delta (Δ) = 26.3 ms, little delta (δ) = 12.2 ms, FOV = 22.4 cm, 2 × 2 × 2 mm^3^ voxels, 60 slices, SENSE reduction factor = 2, number of acquisitions = 1]. The diffusion weighting was isotropically distributed along 32 directions (*b* value = 1000 s/mm^2^). Additionally, three images with no diffusion weighting (*b* value = 0 s/mm^2^) (*b* = 0 images) were acquired using a spin-echo EPI sequence (TR = 10 293 ms, TE = 55 ms, FOV = 22.4 cm, 2 × 2 × 2 mm^3^ voxels, 60 slices). From the collected images, FA maps and MD maps were calculated using the commercially available diffusion tensor analysis package on the MR consol. For more details, see Supplemental Methods.

### Preprocessing of imaging data

Imaging data were preprocessed and analyzed using SPM8 implemented in Matlab. We normalized MD images of subjects with a previously validated (Takeuchi *et al.,* 2013c) diffeomorphic anatomical registration through exponentiated Lie algebra-based registration process method to yield images with 1.5 × 1.5 × 1.5 mm^3^ voxels. Then, tissues that are not likely to be gray or white matter were carefully removed and smoothed by convolving them with an isotropic Gaussian kernel of 8 mm full width at half maximum. For details, see Supplemental Methods.

### Second-level statistical analysis

Using DTI, we investigated the associations of regional MD with individual differences in each TEI scale factor score and total TEI score. Imaging data were analyzed statistically using SPM8. In these analyses, we performed whole-brain multiple regression analyses. In these analyses sex, age, RAPM score, global signal of the analyzed area (defined as the mask created in preprocessing procedures; see Supplemental Methods), and one of the TEI scale factor scores or total TEI score as covariates (resulting in four multiple regression analyses). The analyses of MD were limited to the gray + white matter mask created above. The RAPM score was not significantly correlated with any of the TEI scale factor scores (*P* > 0.05). The global signal was included in the model to eliminate the effects of the global signal that can arise from various types of irrelevant factors (such as the scanner’s noise) as well as to observe regional-specific signals. The variables were mean-centered. The reasons underlying the use of SPM8 in the group-level analyses are described in the Supplemental Methods.

A multiple comparison correction was performed by threshold-free cluster enhancement (TFCE) (Smith and Nichols, 2009) with randomized (5000 permutations) nonparametric testing using the TFCE toolbox (http://dbm.neuro.uni-jena.de/tfce/). We applied a threshold of FWE corrected at *P* < 0.05 (this is according to the custom of the field, but this is a *P* value of one-tailed test).

Although investigation of sex differences in the neural correlates of individual differences in TEI was not included among the primary purpose of this study, we analyzed the interaction effects between sex and TEI scores on MD. No significant results were found. For more details, see the Supplemental Methods and Supplemental Results.

We conducted each analysis of each TEI scale factor score separately because of high correlations among TEI scale factors raise concerns of multicollinearity. However, upon adjustment of other subfactors, the results were observed by performing an additional whole-brain multiple regression analysis, which included three simultaneous TEI scale factors. This revealed minor changes to the results, which did not substantially influence the overall discussion. For these additional analyses and notes, please refer to the Supplemental Methods, Supplemental Results and Supplemental Discussion.

## Results

### Behavioral data

**Fig. 1:**
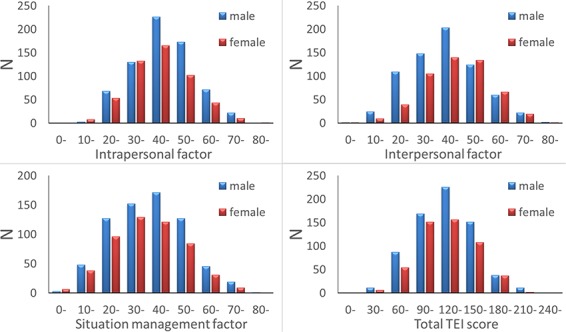
Distribution of scores for each TEI scale factor and the total TEI scale score in our sample.

The average, SD and range of age, RAPM scores, TEI factor scores and total TEI score are presented in Table 1. Distributions of TEI factor scores and total TEI score are presented in Figure 1.

**Table 1:** The average, range and SD of age, RAPM score, scores for each TEI scale factor and the total TEI scale score

Measure	Mean	Range	SD
Age (years)	20.8	18–27	1.8
RAPM	28.5	13–36	3.9
Intrapersonal factor	45.1	12–80	12.1
Interpersonal factor	44.3	5–83	13.5
Situation management factor	39.7	3–80	14.4
Total TEI score	129.1	37–239	34.7

### Associations of regional MD with TEI subfactor scores

**Fig. 2:**
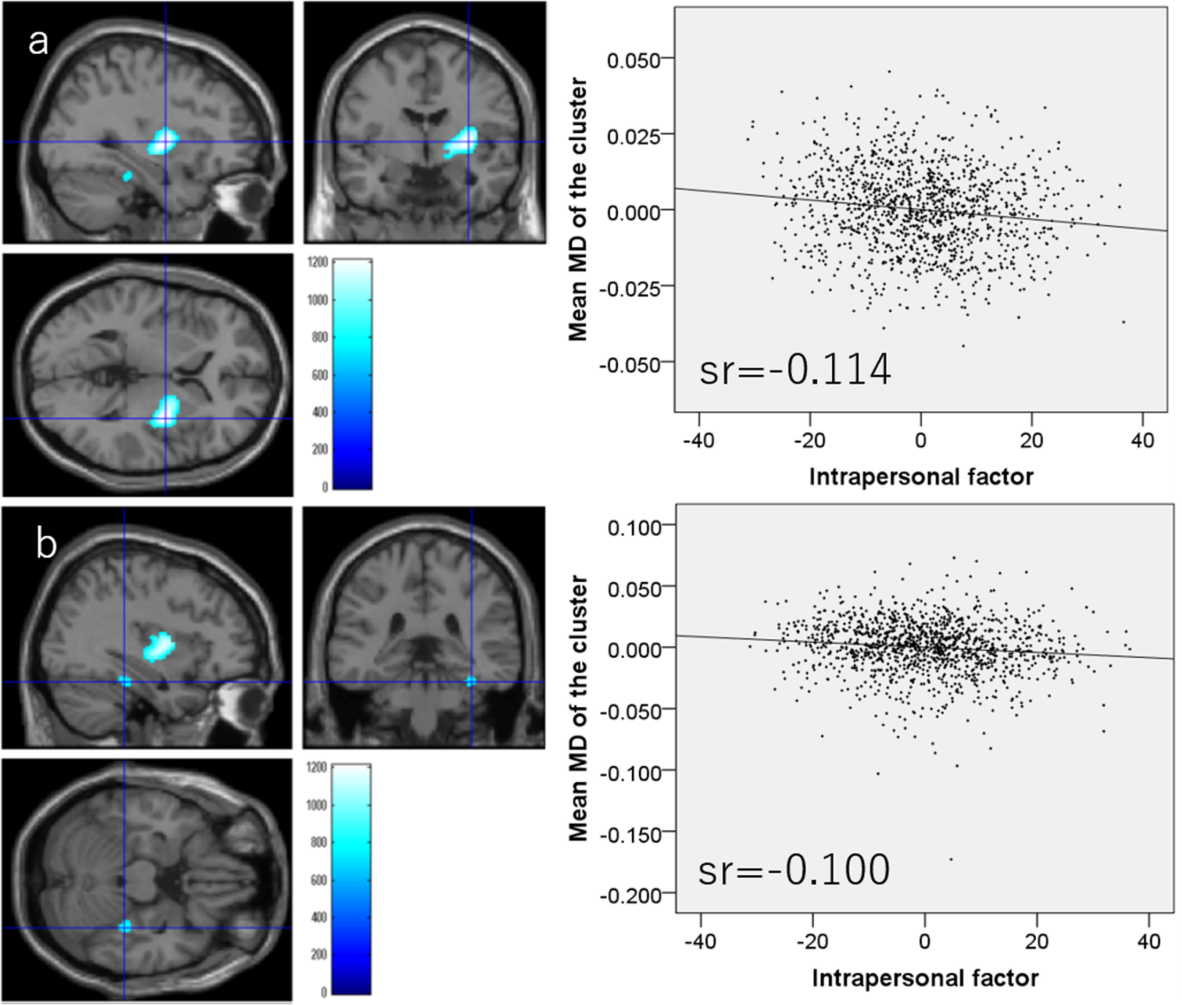
Regions showing significant negative correlations between MD and TEI subfactor scores. (Left panels) The results were obtained using a threshold of TFCE (*P* < 0.05) based on 5000 permutations. Regions with significant correlations are overlaid on a ‘single subject’ T1-weighted image generated by SPM8. The color represents the strength of the TFCE value. (Right panels) Thee right panels show residual plots with trendlines depicting the correlations between residuals in the multiple regression analyses with mean MD in the significant clusters as the dependent variable and other variables as independent variables. Sr represents semi-partial correlation coefficients. (A) Regions showing significant negative correlations between MD and TEI intrapersonal factor scores in and around the right putamen, right globus pallidum and right posterior insula. (B) Regions with significant negative correlations between MD and TEI intrapersonal factor scores are in the right fusiform gyrus.

**Fig. 3:**
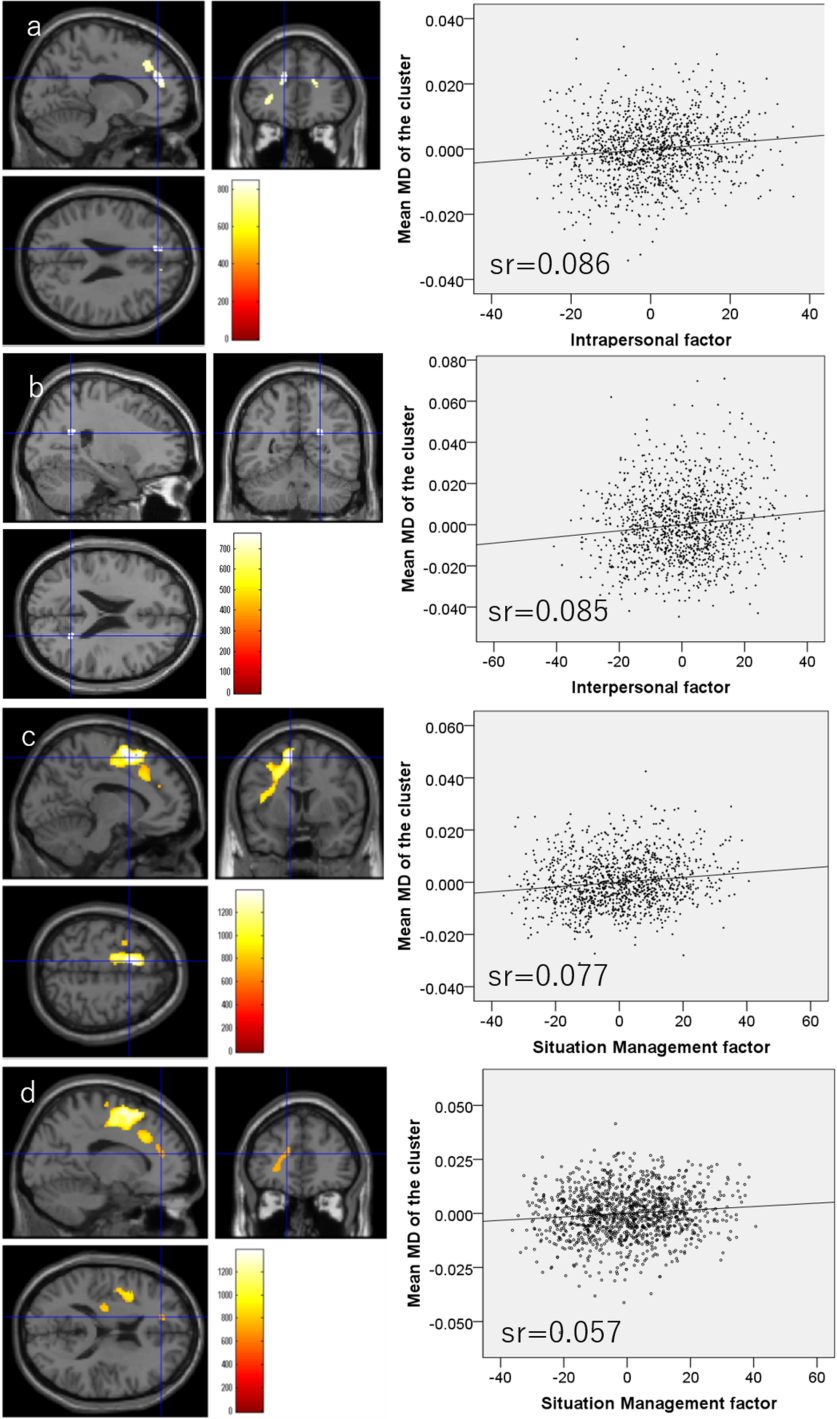
Regions showing significant positive correlations between MD and TEI subfactor scores. (Left panels) The results were obtained using a threshold of TFCE (*P* < 0.05) based on 5000 permutations. Regions with significant correlations are overlaid on a ‘single subject’ T1-weighted image generated by SPM8. The color represents the strength of the TFCE value. (Right panels) The right panels show residual plots with trendlines depicting the correlations between residuals in the multiple regression analyses with mean MD in the significant clusters as the dependent variable and other variables as independent variables. Sr represents semi-partial correlation coefficients. (A) Regions with significant positive correlations between MD and TEI intrapersonal factor scores are mainly distributed around the mPFC, ACC and left inferior frontal gyrus. (B) Regions showing significant positive correlations between MD and TEI interpersonal factor scores were observed in the precuneus. (C) Regions showing significant positive correlations between MD and TEI situation management scores are mainly located around the left ACC, left LPFC and left insula. (D) Regions showing significant positive correlations between MD and TEI situation management scores are also distributed around the left mPFC and left orbitofrontal cortex.

Whole-brain multiple regression analysis revealed that the TEI intrapersonal factor score was significantly and negatively correlated with the MD values of an anatomical cluster that spread in and around the right putamen, right globus pallidum and right posterior insula (Figure 2A) as well as the MD values of a cluster in the right fusiform gyrus (Figure 2B). The analysis also showed that the intrapersonal factor score was significantly and positively correlated with MD values of anatomical clusters distributed mainly in and around areas of the mPFC, ACC and left inferior frontal gyrus (Figure 3A). Further, the whole-brain multiple regression analysis showed that the interpersonal factor score was significantly and positively correlated with MD values in an anatomical cluster located mainly within the precuneus (Figure 3B). Finally, the whole-brain multiple regression analysis showed that the situation management factor score was significantly and positively correlated with MD values of anatomical clusters distributed mainly in and around the anterior cingulate, lateral prefrontal cortex (LPFC) and insula (Figure 3C and D).

All statistical data are presented in Table 2.

**Table 2:** Brain regions exhibiting significant correlations between TEI scale factor scores and MD

Included gray matter areas^*^ (number of significant voxels in the left and right side of each anatomical area)	*x*	*y*	*z*	TFCE value	Corrected *P* value (FWE)	Cluster size (voxel)	Semi-partial correlation coefficients^**^
Negative correlation with intrapersonal factor							
Insula (R:247)/Pallidum (R:446)/Putamen (R:759)/Rolandic operculum (R:7)/Thalamus (R:3)/	33	−4.5	3	1212	0.004	1695	−0.114
Fusiform gyrus (R:101)/	36	−34.5	−22.5	756	0.039	101	−0.100
Positive correlation with intrapersonal factor							
Anterior cingulum (R:103)/Middle frontal other areas (R:1)/Superior frontal medial area (R:57)/Superior frontal other areas (R:46)/	13.5	48	16.5	845	0.022	339	0.086
Superior frontal medial area (L:185)/Superior frontal other areas (L:144)/	−12	40.5	25.5	842	0.023	409	0.070
Inferior frontal orbital area (L:1)/Inferior frontal triangular (L:21)/	−30	39	1.5	750	0.040	105	0.068
Inferior frontal triangular (L:7)/	−39	30	3	718	0.049	7	0.077
Positive correlation with Interpersonal factor							
Cuneus (R:1)/Precuneus (R:41)/	24	−54	22.5	771	0.039	77	0.085
Positive correlation with situation management factor							
Caudate (L:19)/Anterior cingulum (L:2)/Middle cingulum (L:96)/Inferior frontal operculum (L:97)/Middle frontal other areas (L:222)/Superior frontal medial area (L:113)/Superior frontal other areas (L:741)/Insula (L:93)/Paracentral lobule (L:18)/Postcentral gyrus (L:1)/Precentral gyrus (L:142)/Rolandic operculum (L:131)/Supplemental motor area (L:989)/	−10.5	7.5	55.5	1385	0.002	5117	0.077
Anterior cingulum (L:7)/Inferior frontal triangular (L:5)/Superior frontal medial area (L:68)/	−15	42	16.5	738	0.041	276	0.057

^*^The anatomical regions of the gray matter were labeled based on the WFU PickAtlas Tool (http://www.fmri.wfubmc.edu/cms/software#PickAtlas/) (Maldjian *et al.,* 2003, 2004) and on the PickAtlas automated anatomical labeling atlas option (Tzourio-Mazoyer *et al.,* 2002). Temporal pole areas included all subregions in the areas of this atlas.

^**^Semi-partial correlation coefficients of the associations with mean MD values of significant clusters. Note any correlation coefficients in the significant areas of whole brain analyses do not reflect true effect size due to overfitting depending on factors such as sample size and the number of multiple comparisons.

### Associations of MD values with TEI total score

**Fig. 4:**
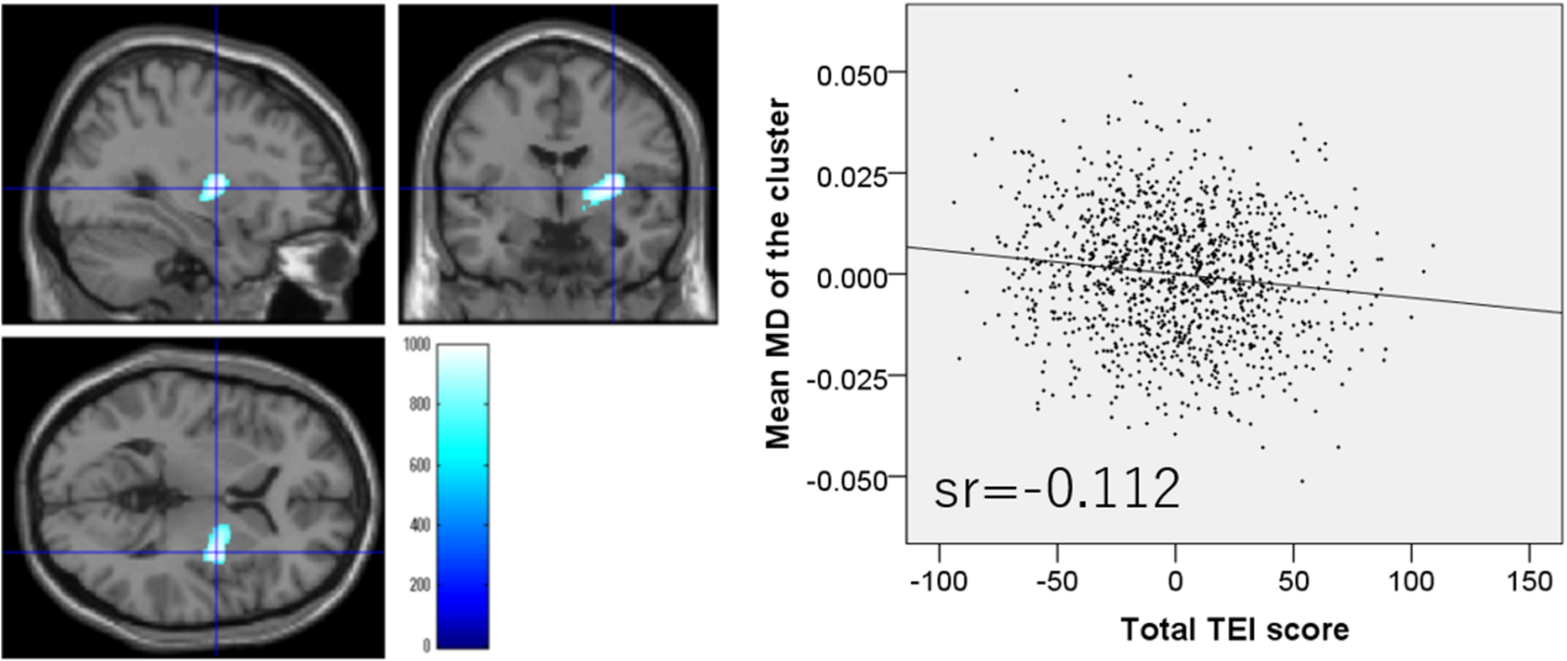
Regions with significant negative correlations between MD values and TEI total score. (Left panel) Results were obtained using a threshold of TFCE (*P* < 0.05) based on 5000 permutations. Regions showing significant correlations are overlaid on a ‘single subject’ T1-weighted image from SPM8. Color represents the strength of the TFCE value. (Right panel) The right panel shows a residual plot with a trendline depicting the correlations between residuals in the multiple regression analyses, with mean MD in the significant clusters as the dependent variable and other variables as independent variables. Sr represents semi-partial correlation coefficients. Regions showing significant negative correlations were seen in and around the right putamen, right globus pallidum and right posterior insula.

**Fig. 5:**
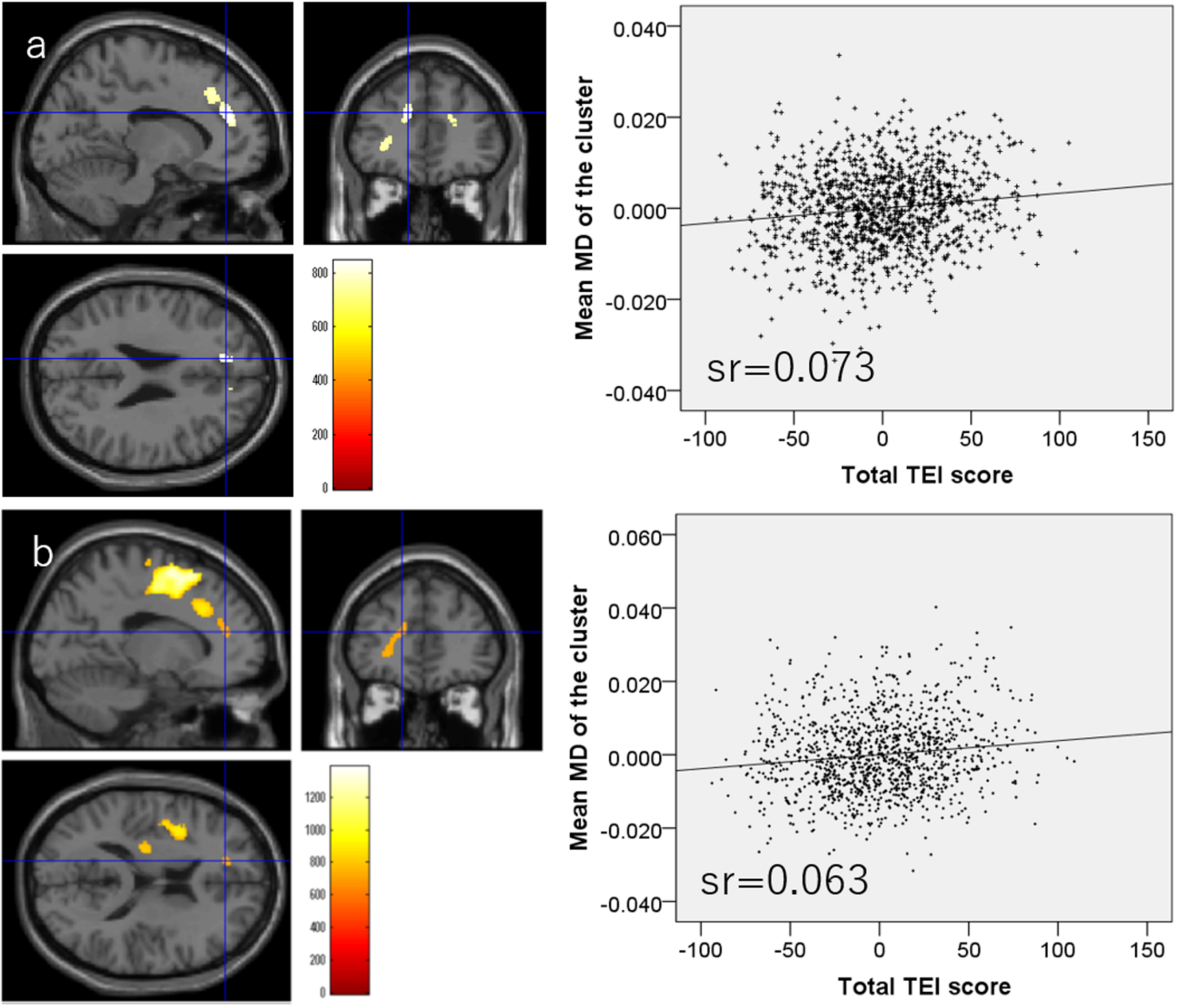
Regions with significant positive correlations between MD values and TEI total score. (Left panels) Results were obtained using a threshold of TFCE (*P* < 0.05) based on 5000 permutations. Regions with significant correlations are overlaid on a ‘single subject’ T1-weighted image from SPM8. Color represents the strength of the TFCE value. (Right panels) The right panels show residual plots with trendlines, depicting the correlation between residuals in the multiple regression analyses and mean MD in the significant clusters, as the dependent variable and other variables as independent variables. (A) Regions of significant positive correlations in anatomical clusters mainly located in the vicinity of the left ACC, left LPFC and left insula. (B) Regions with significant positive correlations in anatomical clusters mainly located around the left mPFC, left orbitofrontal cortex and left inferior frontal gyrus.

Whole-brain multiple regression analysis showed that the total TEI score was significantly and negatively correlated with MD values in an anatomical cluster that spread in and around the right putamen, right globus pallidum and right posterior insula (Figure 4). The analysis also showed total TEI score was significantly and positively correlated with MD values of the anatomical cluster that spread mainly in and around the mPFC, ACC, orbitofrontal gyrus and left inferior frontal gyrus as well as the anatomical cluster that spread in and around the anterior cingulate, LPFC and insula (Figure 5).

All statistical data are presented in Table 3.

**Table 3:** Brain regions exhibiting significant correlations between total TEI scale score and MD value

Included gray matter areas^*^ (number of significant voxels in the left and right side of each anatomical area)	*x*	*y*	*z*	TFCE value	Corrected *p* value (FWE)	Cluster size (voxel)	Semi-partial correlation coefficients^**^
Negative correlation							
Insula (R:73)/Pallidum (R:340)/Putamen (R:432)/Thalamus (R:2)/	31.5	−4.5	4.5	994	0.010	999	−0.112
Positive correlation							
Middle cingulum (L:13)/Inferior frontal operculum (L:70)/Middle frontal other areas (L:2)/Superior frontal medial area (L:4)/Superior frontal other areas (L:249)/Insula (L:25)/Paracentral lobule (L:5)/Precentral gyrus (L:1)/Rolandic operculum (L:47)/Supplemental motor area (L:548)/	−9	9	55.5	951	0.012	1597	0.073
Anterior cingulum (L:11)/Middle cingulum (L:15)/Inferior frontal orbital area (L:5)/Inferior frontal triangular (L:55)/Middle frontal other areas (L:12)/Superior frontal medial area (L:157)/Superior frontal other areas (L:64)/	−30	39	1.5	809	0.029	816	0.063

^*^The anatomical regions of the gray matter were labeled based on the WFU PickAtlas Tool (http://www.fmri.wfubmc.edu/cms/software#PickAtlas/) (Maldjian *et al.,* 2003, 2004) and on the PickAtlas automated anatomical labeling atlas option (Tzourio-Mazoyer *et al.,* 2002). Temporal pole areas included all subregions in the areas of this atlas.

^**^Semi-partial correlation coefficients of the associations with mean MD values of significant clusters. Note any correlation coefficients in the significant areas of whole brain analyses do not reflect true effect size due to overfitting depending on factors such as sample size and the number of multiple comparisons.

## Discussion

In this study, we revealed novel associations between TEI and regional MD values. These results were consistent with our general hypothesis that MDDS and MD values of the areas of the SMC and of the SCN are associated with TEI. However, positive and negative correlations were found, which is in line with the association between better TEI and both greater and smaller rGMD and greater and smaller brain activity (Killgore and Yurgelun-Todd, 2007; Takeuchi *et al.,* 2011). We found that the TEI intrapersonal factor score negatively correlated with MDDS, MD of the areas in and around the right putamen, right pallidum and right posterior insula (consistent with our specific hypothesis 1) as well as with MD values of the right fusiform gyrus, a key node of the SCN. The TEI intrapersonal factor score also positively correlated with MD values of an anatomical cluster that mainly spread in and around the mPFC, a key node of the SCN (consistent with our specific hypothesis 2), the ACC, an important node of the SMC and the left inferior frontal gyrus. We also found that the TEI interpersonal factor score was positively correlated with MD values of the precuneus, a key node of the SCN (consistent with our specific hypothesis 2). Additionally, we found that the TEI situation management factor score was positively correlated with the MD values of an anatomical cluster mainly distributed in and around the anterior cingulate and insula and extended into the vicinity of the orbitofrontal cortex, all of which are important SMC nodes, as well as with the MD values of a cluster around the LPFC. This is partly consistent with our specific hypothesis 3. Finally, the total TEI score was found to be negatively correlated with regional MDDS values of right putamen, right pallidum and right posterior insula. The total TEI score was also found to be positively correlated with MD values of areas around the mPFC, which includes key nodes of the SCN, with areas of the ACC, orbitofrontal gyrus and anterior insula, which are key nodes of the SMC, and with the left inferior frontal gyrus and LPFC. The standardized partial regression coefficient (β) of the associations between TEI scores and mean MD values were around 0.05–0.15, and apparently the effect sizes were small. However, as previously discussed, low effect sizes for the associations between neuroimaging measures and cognitive variables in young adults are a common phenomenon in studies of large samples. This phenomenon is also observed for the associations between representative imaging variables and cognitive variables, such as intelligence and rGMV, and thus is not indicative of the relatively low importance of the observed associations or of the fact that the effect size is lower than that of studies with small sample sizes. See the Supplemental Discussion for further discussion of this issue.

These regions with significant correlations overlapped substantially with the regions defined by our hypotheses. The negative correlations were mainly with the subcortical dopaminergic system, whereas the positive correlations were mainly with areas in or around the neocortex, cingulate and insula. Although the mechanisms are unclear, these association patterns between TEI and MD values are at least partly consistent with our previous studies. And we cited a few possibilities for this explanation. One of our previous studies found that personality traits associated with motivation, such as persistence and novelty seeking, negatively correlated with MDDS. On the other hand, the personality traits that involve pro-social characteristics, such as cooperativeness, positively correlated with MD values of areas within or close to the ACC (Takeuchi *et al.,* 2015b). Another one of our previous studies revealed a negative association between motivational state and MDDS (Takeuchi *et al.,* 2016b), and a submitted study found that empathy positively associated with regional MD in the vicinity of the ACC, LPFC and insula (Takeuchi et al., 2019). Overall, these findings are consistent with our present results in that conditions associated with greater motivation are associated with lower MDDS values, whereas traits associated with social competence or sociality are associated with greater MD values in the anterior and posterior white and gray matter areas. As described in the Introduction, greater tissue density (more numerous cellular elements per unit volume) is believed to prevent free water diffusion and thus lower MD. Therefore, the associations between traits linked to greater motivation, such as the TEI intrapersonal factor, and lower regional MDDS values in areas such as the pallidum and putamen (Takeuchi *et al.,* 2016b) are consistent with this notion. This may also explain the associations between regional MD values of the fusiform area (part of the SCN) (Takeuchi *et al.,* 2013b) and the TEI intrapersonal factor. The positive associations between MD and TEI in the areas of the neocortex and cingulate cortex may be due to the reduced function in some nearby limbic areas associated with emotions, such as anger and anxiety (Seeley *et al.,* 2007), may be associated with greater TEI. However, it appeared that regions of positive associations between MD values and TEI include areas closer to the LPFC and mPFC. Another possibility is that greater MD in regions of significant associations with TEI may reflect increased regional cerebral blood flow at rest (Mancuso *et al.,* 1995) as greater cerebral blood flow has been linked to increased MD value (Jin and Kim, 2008). The SCN overlaps substantially with the default mode network (DMN), which is active during rest and includes regions of the DMN such as the mPFC, precuneus, superior temporal cortex and temporoparietal junction (Fox *et al.,* 2005). In some of these regions, TEI may be associated with greater regional cerebral blood flow during rest in the DMN, which in turn is associated with greater MD value. However, this is speculation and remains to be examined in future studies. The final possibility involves adaptive synaptic pruning during development (Sowell *et al.,* 2003). During development, synaptic pruning leads to reduced gray matter structures (Sowell *et al.,* 2003), and some studies have suggested that advanced cortical thinning underlies intellectual development (Shaw *et al.,* 2006). Further, in our previous studies, we found that TEI factors were associated with reduced regional gray matter density in many areas of the SMC and SCN and suggested that TEI may be associated with advanced cortical thinning during development (Takeuchi *et al.,* 2011). One interesting speculation is that a similar phenomenon may mediate the associations between greater MD values and greater TEI. However, in contrast to the regional gray matter amount decrease during development, MD does not increase substantially during development in white matter (Taki *et al.,* 2013). Moreover, we also found no substantial MD increases in the gray matter of children examined in a previous study (Takeuchi *et al.,* 2016a) (unpublished data). Therefore, this interpretation is not consistent with our findings developmental change in MD and taken cautiously.

The present findings support the notion that dopaminergic system function is important for TEI and suggest that this system is particularly important for the TEI intrapersonal factor (consistent with our specific hypothesis 1). A growing body of evidence suggests the involvement of motivation and of the dopaminergic system that supports motivation in EI. As described in the Introduction, the ability to motivate oneself is an important component of EI (Goleman, 1998; Uchiyama *et al.,* 2001), and EI is also associated with better mood states (Uchiyama *et al.,* 2001) including motivational state (Takeuchi *et al.,* 2015c). The dopaminergic system is an important component of the neural substrate regulating motivation (Carlson, 2001). Further, polymorphisms of the dopamine D2 receptor (DRD2) gene are associated with emotional control, which is an important component of the TEI intrapersonal factor (Blasi *et al.,* 2009) as well as TEI (Takeuchi *et al.,* 2015c). An animal study also showed that dopamine D2 function is important for regulatory self-control (Pattij *et al.,* 2007), an important component of the TEI intrapersonal factor. As described in the Introduction, decreased MDDS is associated with greater motivational state (Takeuchi *et al.,* 2016b), and a number of conditions (diseases, states, and traits) are associated with facilitated dopaminergic function (Takeuchi and Kawashima, in press). A PET study also showed a strong negative correlation between MDDS and dopamine synthesis capacity in some areas of the dopaminergic system (Kawaguchi *et al.,* 2014). Overall, the present neuroimaging findings of robust negative correlations between regional MDDS values and both total TEI and TEI intrapersonal factor scores further support the notion that specific components of the dopaminergic system contribute to TEI. Moreover, the present findings also advance our understanding of dopaminergic mechanisms of TEI as previous studies using morphometry or structural and functional connectivity analyses generally failed to find significant associations of TEI with the dopaminergic system (Takeuchi *et al.,* 2011, 2013a,b). However, it should be noted that, upon adjusting other subfactors, the correlation between MD values in the right putamen and pallidum and TEI intrapersonal factor was no longer significant (although the left homologue showed a significant negative correlation, see Supplemental Results). MD in this area may have certain relevance to other TEI subfactors as well.

In addition, we found that TEI intrapersonal and interpersonal factors were associated with MD of the medial cortical areas in proximity of the SCN (consistent with our specific hypothesis 2), suggesting the functional involvement of these areas in TEI. As described above, given the specific regional patterns of the associations, we speculate that the positive correlations of MD values in the DMN with TEI score could reflect associations between greater TEI and higher function or activity within these areas due to increased blood flow (or activity) at rest. Regional MD values in the vicinity of the mPFC were significantly and positively correlated with the TEI intrapersonal factor as well as total TEI score. The mPFC is a key node of the SCN and is involved in the perception of self (self-knowledge) as well as cognition regarding others (person perception mentalizing, which refers to our ability to read the mental states of other agents) (Amodio and Frith, 2006). Further, regional MD in the vicinity of the precuneus was significantly and positively correlated with the TEI interpersonal factor. This area, which is also a key node of SCN, is involved in a wide range of cognitive functions associated with the self and others. Such functions include self-relevant information processing, self-awareness and representation of the mental self as well as the emotional state attribution of others, social cognition, perspective taking and mental stimulation of others (Cavanna and Trimble, 2006). Note that both of these areas are involved in self-related cognition as well as cognition related to others, consistent with the notion that neural mechanisms of self-cognition and social cognition overlap (Uddin *et al.,* 2007). These findings support our hypothesis that areas of the SCN are involved in TEI. Although MD of the mPFC is only associated with the intrapersonal factor score and MD of the precuneus only with the interpersonal factor score at the applied threshold, neural mechanisms within both areas may be associated with TEI intrapersonal and interpersonal factors. Indeed, our previous study (Takeuchi *et al.,* 2011) showed that regional gray matter density in the precuneus negatively correlated with TEI intrapersonal factor score and resting-state functional connectivity with mPFC significantly and positively correlated with TEI interpersonal factor score (Takeuchi *et al.,* 2013a). The TEI intrapersonal factor score was also significantly and negatively correlated with MD of the fusiform gyrus. This area is involved in face recognition along with other cognitive functions (for a review, see Kanwisher and Yovel, 2006) and is regarded as a subpart of the SCN (Takeuchi *et al.,* 2013b). We previously showed that the white matter structure connected to the fusiform gyrus is positively correlated with TEI interpersonal factor (Takeuchi *et al.,* 2013b). However, this area is also involved in self-face recognition (Sugiura *et al.,* 2005) and self-reference processing, regardless of the type of stimulus (Kircher *et al.,* 2000). Moreover, upon adjusting for other TEI subfactors, the significant correlation between the TEI intrapersonal factor and MD in this area was no longer observed (see Supplemental Results). Perhaps, this area may also play a key role in both self-cognition and cognition of others and contribute to both the TEI intrapersonal and interpersonal factors.

Further, our findings showed that the TEI situation management factor and total TEI score were associated with regional MD values in the vicinity of the ACC and insula (partly consistent with our specific hypothesis 3). Furthermore, the total TEI score was associated with regional MD values in the vicinity of the orbitofrontal cortex and VMPFC. Among regions of the SMC, the VMPFC is assumed to be particularly important for EI as it uses integrated somatic markers to affect decision making toward advantageous outcomes (Bechara and Damasio, 2005). Further, among regions of the SMC, studies suggest that the insula plays a key role in monitoring ongoing somatic and visceral states (Reiman, 1997; Reiman *et al.,* 1997). Finally, the ACC is also regarded as a key node of the salience network, which includes the anterior insula, another key node of the SMC, as well as the ACC (Seeley *et al.,* 2007; Taylor *et al.,* 2009). This network is thought to integrate interoceptive information with emotional salience and contributes to the SMC (Taylor *et al.,* 2009). Therefore, if greater MD values of these areas reflect greater cerebral blood flow, neural activity or some other regional process, these findings are consistent with our hypothesis that TEI is associated with regional MD of SMC areas. However, as discussed above, greater MD value may be reflective of less tissue in these areas, or these areas may be associated with TEI through other mechanisms. For example, the salience network or areas of the anterior insula and ACC are implicated in negative emotions such as anger and anxiety (Seeley *et al.,* 2007; Takeuchi *et al.,* 2014a), and lower functioning of these areas may be important for TEI (or greater TEI may suppress activity in regions associated with negative emotions). Future studies are needed to test these ideas. Additionally, TEI intrapersonal factors showed similar correlations in contingent areas. Upon adjustment for other subfactors, the areas of the correlations of TEI situation management factor became substantially limited (Supplemental Results). The specificity of such associations may not be clear.

Finally, we found that significant correlation between MD and TEI extended into regions close to the dorsolateral prefrontal cortex and inferior frontal gyrus. Areas of significant correlations between MD values and TEI intrapersonal factor score as well as between MD and the total TEI score also extended into areas adjacent to the inferior frontal gyrus. Further, the areas of significant correlations between MD and TEI situation management factor score as well as between MD value and total TEI score extended into areas adjacent to the dorsolateral prefrontal cortex. Among many functions of the left inferior frontal gyrus, inhibition of unwanted responses may be particularly relevant to EI (Aron *et al.,* 2004). Further, the dorsolateral prefrontal cortex is suggested to play a key role in human intelligence and central executive functions (Duncan *et al.,* 2000; Baddeley, 2003). Given the broad concept of EI, these traditional intellectual abilities may also contribute to EI. Perhaps, the associations of TEI with MD values found in this study reflect associations of these cognitive functions with TEI. TEI has been shown to be more closely aligned with personality constructs, whereas AEI correlates more strongly with traditional intelligence metrics (Copestake *et al.,* 2013; Webb *et al.,* 2013, 2014; Vesely Maillefer *et al.,* 2018). Notably, RAPM score did not show a significant relationship with TEI scores. However, additional partial regression analyses using the cognitive measures available in this project suggested significant associations between TEI measures and some speeded cognitive measures such as the Tanaka B-type intelligence test (TBIT) type 3B (Tanaka *et al.,* 2003). TBIT measures psychometric intelligence based on speeded cognitive tasks. After adjusting for age and sex, TBIT showed a significant positive association with the intrapersonal TEI subfactor score (partial regression coefficient β = 0.067, *P* = 0.026), the Situation Management subfactor score (β = 0.092, *P* = 0.003) and the total TEI score (β = 0.079, *P* = 0.009) (*N* = 1085). These results support the abovementioned interpretation in the present study setting. However, areas of the LPFC are associated with a wide range of functions, including reverse inference, so future studies are needed to examine these notions.

This study has certain few limitations. One is the inclusion of only highly educated young adults from a developed country, traits common to most similar studies (Jung *et al.,* 2010; Takeuchi *et al.,* 2015b; Yang *et al.,* 2016). Therefore, whether our findings can be generalized to other populations must be confirmed. Another limitation is the focus on only TEI. Although we focused on TEI for the reasons described in the Introduction, previous studies have reported that AEI, rather than TEI, is associated with neural mechanisms (Killgore *et al.,* 2013, 2017). Future studies are warranted to reveal the MD correlates of AEI. As described in Supplemental Methods, previous studies have shown an association between the Emotional Intelligence Scale and other conceptually relevant laboratory psychological measures. However, to our knowledge, studies showing the association between Emotional Intelligence Scale and measures outside the laboratory (in a real-world setting) remain scarce. A previous study has shown that students who received employment offer(s) show significantly higher scores in all three TEI subfactors than those who did not (Shimai *et al.,* 2007). Future studies are required to accumulate more evidence of the ecological validity of the emotional intelligence scale.

In summary, this is the first study to investigate the associations of TEI with regional MD. The TEI intrapersonal factor and total TEI scores were negatively associated with MDDS, thereby providing neuroimaging evidence for the importance of the dopaminergic system in TEI. Our findings also revealed positive associations between TEI and regional MD values in lateral and medial cortical structures of the SMC and SCN as well as lateral prefrontal areas. Together with previous study findings using other neuroimaging techniques, our findings may further support the importance of SCN- and SMC-related neural mechanisms in TEI. Moreover, they also demonstrate the importance of lateral prefrontal cortices to TEI as well as possible associations of TEI with other traditional cognitive domains. However, the mechanisms underlying positive associations (elevated MD) are still unclear. Future studies using additional experimental techniques are needed to reveal the neural mechanisms underlying these correlation patterns.

## Ethical statement

- Compliance with Ethical Standards: All procedures performed in studies involving human participants were in accordance with the ethical standards of the institutional and/or national research committee and with the 1964 Helsinki declaration and its later amendments or comparable ethical standards.
- Ethical approval: This study was approved by the Ethics Committee of Tohoku University.
- Informed consent: Informed consent was obtained from all individual participants included in the study.

## Funding

This study was supported by JST/RISTEX, JST/CREST, a Grant-in-Aid for Young Scientists (B) (KAKENHI 23700306) and a Grant-in-Aid for Young Scientists (A) (KAKENHI 25700012) from the Ministry of Education, Culture, Sports, Science, and Technology.

## Conflict of interest

The authors declare that they have no conflict of interest.

## Supplementary Material

scan-18-395-File007_nsz059
