# Supplementary material for: Mean diffusivity associated with trait emotional intelligence
Source: Soc Cogn Affect Neurosci. 2019 Oct 8;14(8):871–83. doi: 10.1093/scan/nsz059 (PMC6847659; doi:10.1093/scan/nsz059)
Supplement: scan-18-395-File007_nsz059 [file scan-18-395-file007_nsz059.docx]

**Supplemental online material**

**Supplemental Methods**

**Subjects.** The present study—a part of an ongoing project to investigate the associations among brain imaging findings, cognitive functions, and aging—included TEI measures and imaging data from 1207 healthy, right-handed individuals (693 men and 514 women). The mean age of the subjects was 20.8 years [standard deviation (SD), 1.8; age range: 18–27 years old]. The following descriptions are reproduced mainly from one of our previous studies of the same ongoing project using the same methods ([Hikaru Takeuchi et al., 2015a](#_ENREF_22)). All subjects were university students, postgraduates, or university graduates of <1 year’s standing. All subjects had normal vision and none had a history of neurological or psychiatric illness. Handedness was evaluated using the Edinburgh Handedness Inventory ([Oldfield, 1971](#_ENREF_12)). Written informed consent was obtained from each subject. For non-adult subjects, written informed consent was obtained from their parents or guardians. This study was approved by the Ethics Committee of Tohoku University.

Subjects were instructed to get sufficient sleep, maintain their regular schedule, eat sufficient breakfast, and consume their normal amounts of caffeinated foods and drinks in the day of cognitive tests and magnetic resonance imaging (MRI) scans. In addition, subjects were instructed to avoid alcohol the night before the assessment.

**The information of validity and reliability of Emotional Intelligence Scale.** The Emotional Intelligence Scale is an established test based on normative data with a large sample size (n = 703) ([Uchiyama et al., 2001](#_ENREF_34)). The scoring of each factor is based on a test manual. Confirmatory factor analyses have validated the three-component model of this test ([Otake et al., 2001](#_ENREF_14); [Uchiyama et al., 2001](#_ENREF_34)). According to the test manual ([Uchiyama et al., 2001](#_ENREF_34)), the internal consistencies of the three factors (intrapersonal, interpersonal, and situation management) as expressed by Cronbach’s coefficient alpha are 0.894, 0.915, and 0.915, respectively.

As summarized in our previous study ([H. Takeuchi et al., 2011a](#_ENREF_27); [H. Takeuchi et al., 2013b](#_ENREF_29)), in addition to the three-component model of TEI, four- ([Salovey & Mayer, 1990](#_ENREF_16)) and five-component models ([Bar-On, 1997](#_ENREF_1)) are available. The Bar-On model of TEI ([Bar-On, 1997](#_ENREF_1)) consists of two major factors, an intrapersonal and an interpersonal factor, in addition to other minor factors, such as stress coping, adaptability, and general mood. On the contrary, Otake et al. ([2001](#_ENREF_14)) proposed a third major factor (situation management), which is equivalent to the minor factors of the Bar-On model.

Scores on the Emotional Intelligence Scale are associated with other EI-related measurements such as the Toronto Alexithymia Scale ([Fukunishi et al., 2001](#_ENREF_5)), supporting its external validity. All three factors of the Emotional Intelligence Scale are associated with improved mental health as determined by a general health questionnaire as well as increased optimism as determined by the LOT Optimism scale ([Uchiyama et al., 2001](#_ENREF_34)). Specifically, the situation management factor score was strongly associated with better mental health ([Uchiyama et al., 2001](#_ENREF_34)), consistent with the idea that TEI leads to better mental health ([Salovey et al., 2000](#_ENREF_17)).

**Psychometric measures of general intelligence**

The Raven’s Advanced Progressive Matrix (RAPM) ([Raven, 1998](#_ENREF_15)) is considered a relatively pure measure of fluid reasoning ([Raven, 1998](#_ENREF_15)). The following descriptions are reproduced mainly from one of our previous studies using the same methods ([Hikaru Takeuchi et al., 2013a](#_ENREF_21)). Although there are numerous measures of intelligence, and previous imaging studies of intelligence have used a variety of intelligence measures, we used the RAPM due to its strong association with general intelligence and ease of use. In the current study, we used the RAPM to assess intelligence and to adjust for the effect of general intelligence on regional brain structure and also to eliminate the possibility that the associations between EI and mean diffusivity are explained by the combined associations between general intelligence and brain structures as well as between general intelligence and EI. RAPM scores were adjusted according to our previous studies on the neural bases of individual EI ([Hikaru Takeuchi et al., 2013a](#_ENREF_21); [H. Takeuchi et al., 2011a](#_ENREF_27); [H. Takeuchi et al., 2013b](#_ENREF_29)). More detailed information on how this test was performed in the present study can be found in our previous studies ([H. Takeuchi et al., 2010b](#_ENREF_25), [2010c](#_ENREF_26)).

**Additional details of diffusion image acquisition.** We also acquired data for phase correction and signal stabilization not used as reconstructed images. Mean diffusivity (MD) and FA maps were calculated from the collected images using a commercially available diffusion tensor analysis package on the MR consol. This methodology has been used in many of our previous studies ([H. Takeuchi et al., 2010a](#_ENREF_19); [H. Takeuchi et al., 2010c](#_ENREF_26), [2011b](#_ENREF_28); [H. Takeuchi et al., 2013b](#_ENREF_29); [Hikaru Takeuchi et al., 2013c](#_ENREF_31)). Furthermore, the results of analyses using these images were consistent with those of previous studies using alternative methods ([Barnea-Goraly et al., 2005](#_ENREF_2); [Taki et al., 2013](#_ENREF_32)), suggesting the validity of the current techniques. These procedures involved correction for motion and distortion caused by eddy currents. Calculations were performed according to a previously proposed method ([Le Bihan et al., 2001](#_ENREF_7)). These descriptions are reproduced mainly from our previous study using the same methods ([Hikaru Takeuchi et al., 2016](#_ENREF_20)).

**Preprocessing of imaging data**

Preprocessing and analysis of diffusion imaging data were performed using SPM8 implemented in Matlab. Most of the following descriptions were reproduced from our previous study using the same methods ([Hikaru Takeuchi et al., 2016](#_ENREF_20)). First, the skull in the mean b = 0 image of each participant was stripped as described previously ([H. Takeuchi et al., 2010c](#_ENREF_26)); using the resulting image, diffusion images were linearly aligned with the previously created skull-stripped b = 0 image template ([H. Takeuchi et al., 2010c](#_ENREF_26)) to assist with the following procedures.

Subsequently, using a previously validated new two-step segmentation algorithm of diffusion images and the previously validated diffeomorphic anatomical registration through exponentiated lie algebra (DARTEL)-based registration process that utilized the information of the FA signal distribution within the white matter tissue ([for details, see Hikaru Takeuchi et al., 2013c](#_ENREF_31)), all diffusion images were normalized. These normalized images included gray matter segments [regional gray matter density (rGMD) map], white matter segments [regional white matter density (rWMD) map], and cerebrospinal fluid (CSF) segments [regional CSF density (rCSFD) map]. The voxel size of these normalized images was 1.5 × 1.5 × 1.5 mm^3^. For these processes, we used the DARTEL template that we created in our previous study from subjects participating in the same project ([for details, see Hikaru Takeuchi et al., 2013c](#_ENREF_31)).

Next, we created average images from the normalized rGMD and rWMD images of all subjects whose diffusion imaging data were obtained in the pre-experiment. Subsequently, for the analyses of MD images from the normalized images of the (a) MD, (b) rGMD, and (c) rWMD maps, we created MD images from which areas not likely to be gray or white matter were removed. Removed areas were defined as “gray matter tissue probability + white matter tissue probability < 0.99” in our normalized rGMD and rWMD maps. These removals were conducted to exclude the strong effects of CSF on MD throughout analyses. These normalized masked MD images were then smoothed (8-mm full-width half-maximum) and carried through to the second-level analyses of MD.

We did not use T1-weighted structural images for normalization and calculation of GMC and WMC maps for correction because T1-weighted structural and EPI images have apparent differences due to the distortion caused by 3T MRI. Therefore, T1-weighted structural images is apparently not suited for the accurate and precise segmentation and normalization of images for MD maps.

We used SPM8, and not SPM12, for these procedures because the methods and parameters were optimized and validated in our previous study ([Hikaru Takeuchi et al., 2013c](#_ENREF_31)) using SPM8.

**Effects of interaction between sex and TEI scores on regional MD**

To investigate if the MD correlates of TEI scores differed between sexes, we performed whole-brain analyses of covariance (ANCOVAs). The dependent variables in these analyses were same as those in the whole-brain multiple regression analyses that were conducted to investigate the correlation with TEI scores across sexes and MD values in each voxel. In these whole-brain ANCOVAs, sex was a group factor (using the full factorial option in SPM8), whereas age, RAPM score, global signal of the analyzed area, and one of the TEI scale factor scores or total TEI score were covariates (resulting in four whole-brain ANCOVAs). In addition, age, RAPM score, and TEI scores were modeled to enable unique relationships with MD (using the interactions option in SPM8) for each sex. The global signal of the analyzed area was modeled to have common relationships with MD across sexes. The interaction effects between sex and TEI score (one TEI scale factor score or total TEI score) were assessed using t-contrasts. Correction for multiple comparisons was performed using the same method used in the whole-brain multiple regression analyses.

**Reasons underlying use of SPM8 in group level statistical analyses**

We used SPM8 for statistical analyses because of the compatibility of the software used for permutation-based statistics and the home-made script used for the statistical analyses. If permutation tests are used, the results should not be affected by the version of SPM.

**Whole-brain multiple regression analysis including three TEI scale factor scores as covariates simultaneously.**

In this study, r = 0.549 for the correlation between the intrapersonal factor score and the interpersonal factor score; r = 0.720 for the correlation between the intrapersonal factor score and the situation management factor score; r = 0.599 for the correlation between the interpersonal factor score and the situation management factor score. These correlation coefficients were almost as high as correlations coefficients between verbal IQ and performance IQ (r ≒ 0.7) obtained in the Wechsler IQ test ([Wechsler, 1997](#_ENREF_36)). These high correlation coefficients raise concerns of multicollinearity if the three subfactors are added in a multiple regression analysis. Dropping highly correlated variables is suggested as a simple effective strategy against this problem ([Duzan & Shariff, 2015](#_ENREF_4)). However, results were observed upon adjustment of other variables, by performing an additional multiple regression analysis including three TEI scale factors simultaneously.

In this analysis, sex, age, RAPM score, global signal of the analyzed area (defined as the mask created in preprocessing procedures, as described above), and the three TEI scale factor scores were used as covariates. Other statistical procedures (analyzed areas, correction for multiple comparisons) were as described in the main text.

**Supplemental Results**

**Effects of interaction between sex and TEI score on regional MD**

There were no significant effects of interaction between sex and TEI score (TEI scale factor score or total TEI score) in the whole-brain analyses.

**Results of a whole-brain multiple regression analysis including three TEI scale factor scores as covariates simultaneously.**

Whole-brain multiple regression analysis revealed a significant negative correlation between the TEI intrapersonal factor score and the MD values of an anatomical cluster that spread in and around the left putamen and left globus pallidum (Supplemental Fig. 1). The areas showing significant correlations of TEI intrapersonal factor scores in the main text spread in the right putamen, right globus pallidum, and right insula and did not show significance in this supplemental analysis although a tendency was found (P = 0.087, corrected for multiple comparisons, permutation using TFCE scores). The areas showing significant correlations of TEI intrapersonal factor scores in the main text that spread in the right fusiform gyrus showed neither significance nor a tendency (P > 0.15, corrected) in this supplemental analysis.

The analysis also showed a significant positive correlation between the intrapersonal factor score and the MD values of the anatomical clusters distributed mainly in and around areas of the mPFC and the dorsal part of the mPFC (Supplemental Fig. 2a). These significance areas were similar to those showing a significant correlation between MD and intrapersonal factor scores in the main analysis, although the area of the former was limited.

Whole-brain multiple regression analysis showed a significant positive correlation between the interpersonal factor score and MD values in an anatomical cluster located mainly within the precuneus as well as in the lateral right temporoparietal areas (Supplemental Fig. 2b). The precuneus areas showing significance in this supplemental analysis were similar to those showing a significant correlation between MD and interpersonal factors scores in the main analysis.

Finally, whole-brain multiple regression analysis showed significant positive correlation between the situation management factor score and the MD values of anatomical clusters distributed mainly in and around the anterior cingulate and lateral prefrontal cortex (LPFC) (Supplemental Fig. 2c). These significance areas were similar to those showing a significant correlation between MD and situation management factor scores in the main analysis, although the areas of the former were limited.

All statistical data are presented in Supplemental Table 1.

**Supplemental Discussion**

**Low effect sizes of the associations between TEI scores and MD**

In the present study, the strength of the standardized partial regression coefficients (β) of the associations between TEI score and MD were approximately 0.05–0.15, and apparently the effect size is not large or medium. As discussed in our previous studies ([H Takeuchi et al., 2018](#_ENREF_23)), weak correlations (r < 0.2) between individual cognitive differences and neuroimaging measures are very common in studies of large (N > several hundred) samples of typical young adults regardless of the specific imaging or cognitive measures ([Magistro et al., 2015](#_ENREF_8); [Schilling et al., 2012](#_ENREF_18); [Hikaru Takeuchi et al., 2015a](#_ENREF_22); [Hikaru Takeuchi et al., 2017](#_ENREF_24)). In fact, we are not aware of any exceptions. A similar phenomenon is observed for associations between representative imaging and psychological measures, such as the associations between psychometric intelligence and regional gray matter structures or brain activity during working memory ([Schilling et al., 2012](#_ENREF_18); [H Takeuchi et al., 2018](#_ENREF_23); [Hikaru Takeuchi et al., 2017](#_ENREF_24)). Thus, the weak correlations observed in this study are not reflective of the relatively low importance of the observed associations in the field. The large effect sizes and correlation coefficients for peak voxels in significant areas yielded by whole-brain analyses of small samples do not indicate true effect sizes or correlation strengths ([e.g., Murphy et al., 2012](#_ENREF_11)). Rather, in whole-brain analyses—especially those with small samples—the effect sizes were overestimated due to overfitting ([Vul et al., 2009](#_ENREF_35)). In addition, due to publication biases and stringent thresholds, studies of whole-brain analyses with small sample sizes cannot report significant findings of small effect size. Even when single studies show remarkable effect sizes (especially under low statistical power), the effect size reported in meta-analyses can be very small ([e.g., Murphy et al., 2012](#_ENREF_11)). Considering these observations, we cannot presume a large effect size in such studies with small sample sizes. Further, as previously discussed, neuroscience studies typically suffer from low sample size and low statistical power, which may result in overestimation of effect size and lower replicability in the field ([Button et al., 2013](#_ENREF_3)). As problems of low replicability exist in multiple fields ([Open-Science-Collaboration, 2015](#_ENREF_13)), we believe that it is very important to report robust statistical results using large sample size and robust statistical methods.

**Discussion of the results of whole-brain multiple regression analysis including three TEI scale factor scores as covariates simultaneously.**

As described in the Supplemental Results, the areas in the main analysis results were similar to those in the supplemental online material. However, the significant negative correlation between MD in the right fusiform gyrus and the intrapersonal factor scores in the main text became insignificant in the supplemental analysis, in which interpersonal factor scores and Situation management factors were adjusted. This may be due to an association between this area and components of other TEI subfactors such as interpersonal communication, since this area plays a key role in face processing ([for a review, see Kanwisher & Yovel, 2006](#_ENREF_6)). Moreover, the negative correlation between intrapersonal factor scores and MD observed in the right putamen and adjacent areas in the main text became insignificant with only a tendency in the supplemental analysis, in which both interpersonal and situation management factor scores were adjusted. Instead, in the latter analysis, the MD of the right homologue showed a significant negative correlation with the intrapersonal factor scores. The implications of these changes are not clear. In our previous study, personality traits such as lower harm avoidance and higher self-directedness were associated with a lower MD of the right putamen and adjacent areas ([Hikaru Takeuchi et al., 2015b](#_ENREF_30)). Persistence was associated with a lower MD of the left putamen and adjacent areas ([Hikaru Takeuchi et al., 2015b](#_ENREF_30)). All these personality traits were associated with the motivational state ([Hikaru Takeuchi et al., 2015b](#_ENREF_30)). However, each personality has different characteristics and that adjustment of other TEI subfactor scores may alter the nature of correlation of TEI intrapersonal factors.

**Supplemental Table 1**

Brain regions exhibiting significant correlations between TEI scale factor scores and MD in the whole-brain multiple regression analysis including three simultaneous TEI scale factor scores as covariates

| Included gray matter areas* (number of significant voxels in the left and right sides of each anatomical area) | x | y | z | TFCE value | Corrected P value (FWE) | Cluster size (voxel) | Semi-partial correlation coefficients** |
| --- | --- | --- | --- | --- | --- | --- | --- |
| Negative correlation with intrapersonal factor |  |  |  |  |  |  |  |
| Pallidum (L:54)/Putamen (L:163) | −25.5 | −6 | 4.5 | 765.05 | 0.038 | 213 | −0.105 |
| Positive correlation with intrapersonal factor |  |  |  |  |  |  |  |
| Anterior cingulum (R:28)/Superior frontal medial area (R:110)/Superior frontal other areas (R:43) | 12 | 49.5 | 16.5 | 823.98 | 0.030 | 192 | 0.102 |
| Positive correlation with Interpersonal factor |  |  |  |  |  |  |  |
| Calcarine Cortex (R:1)/Cuneus (R:3)/Precuneus (R:73) | 24 | −52.5 | 22.5 | 846.36 | 0.021 | 364 | 0.081 |
| Angular gyrus (R:37)/Supramarginal gyrus (R:85)/Superior temporal gyrus (R:7) | 48 | −45 | 25.5 | 788.78 | 0.032 | 161 | 0.089 |
| Angular gyrus (R:1)/Middle occipital lobe (R:39)/Middle temporal gyrus (R:10) | 40.5 | −69 | 22.5 | 739.31 | 0.042 | 74 | 0.082 |
| Angular gyrus (R:48)/Middle temporal gyrus (R:2) | 43.5 | −58.5 | 28.5 | 725.76 | 0.043 | 51 | 0.081 |
| Positive correlation with situation management factor |  |  |  |  |  |  |  |
| Superior frontal other areas (L:199)/Supplemental motor area (L:242) | -10.5 | 1.5 | 63 | 822.23 | 0.029 | 502 | 0.079 |
| Middle frontal other areas (L:32)/Precentral gyrus (L:24) | −31.5 | 6 | 46.5 | 738.28 | 0.045 | 57 | 0.071 |
| None | −28.5 | 1.5 | 36 | 724.35 | 0.049 | 20 | 0.070 |

*The gray matter’s anatomical regions were labeled based on the WFU PickAtlas Tool (<http://www.fmri.wfubmc.edu/cms/software#PickAtlas/>) ([Maldjian et al., 2004](#_ENREF_9); [Maldjian et al., 2003](#_ENREF_10)) and on the PickAtlas automated anatomical labeling atlas option ([Tzourio-Mazoyer et al., 2002](#_ENREF_33)). Temporal pole areas included all subregions in the areas of this atlas.

**Semi-partial correlation coefficients of the associations with mean MD values of significant clusters. Note that any correlation coefficients in significant areas of whole-brain analyses do not reflect a true effect size due to overfitting depending on factors such as sample size and the number of multiple comparisons.

**Supplemental figure legends.**

**Supplemental Fig. 1.** The region showing significant negative correlations between MD and TEI intrapersonal factor scores in whole-brain multiple regression analysis including three TEI subfactor scores as covariates simultaneously. Significant correlations were found in the area of the left globus pallidus and left putamen. (left panel) The results were obtained using a threshold of threshold-free cluster enhancement (TFCE, *P* < 0.05) based on 5000 permutations. Regions with significant correlations are overlaid on a “single subject” T1-weighted image generated by SPM8. The color represents the strength of the TFCE value. (Right panel) The right panel showed residual plots with trendlines depicting the correlations between residuals in the multiple regression analyses with mean MD in the significant clusters as the dependent variable and other scales as independent variables. Sr represents semi-partial correlation coefficients.

**Supplemental Fig. 2.** Regions showing significant positive correlations between MD and trait emotional intelligence (TEI) subfactor scores in whole-brain multiple regression analysis including three TEI subfactor scores as covariates simultaneously. (left panels) The results were obtained using a threshold of threshold-free cluster enhancement (TFCE, *P* < 0.05) based on 5000 permutations. Regions with significant correlations are overlaid on a “single subject” T1-weighted image generated by SPM8. The color represents the strength of the TFCE value. (Right panels) Right panels showed residual plots with trendlines depicting the correlations between residuals in the multiple regression analyses with mean MD in the significant clusters as the dependent variable and other scales as independent variables. Sr represents semi-partial correlation coefficients. (a) Regions showing significant positive correlations between MD and TEI intrapersonal factor scores are mainly distributed around mPFC and ACC. (b) Regions showing significant positive correlations between MD and TEI interpersonal factor scores are seen in the precuneus area and areas around the right temporoparietal junction. (c) Regions showing significant positive correlations between MD and TEI situation management scores are mainly located around the left ACC and left LPFC.

**Supplemental Fig. 1.**


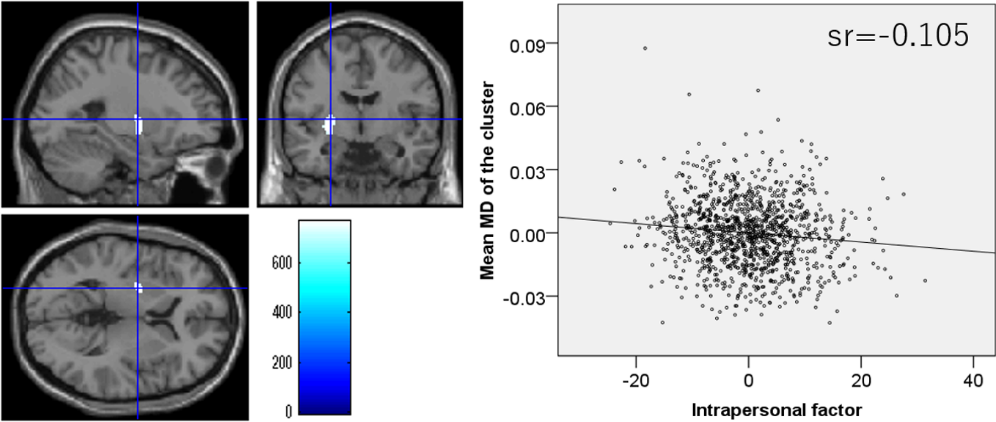


**Supplemental Fig. 2.**


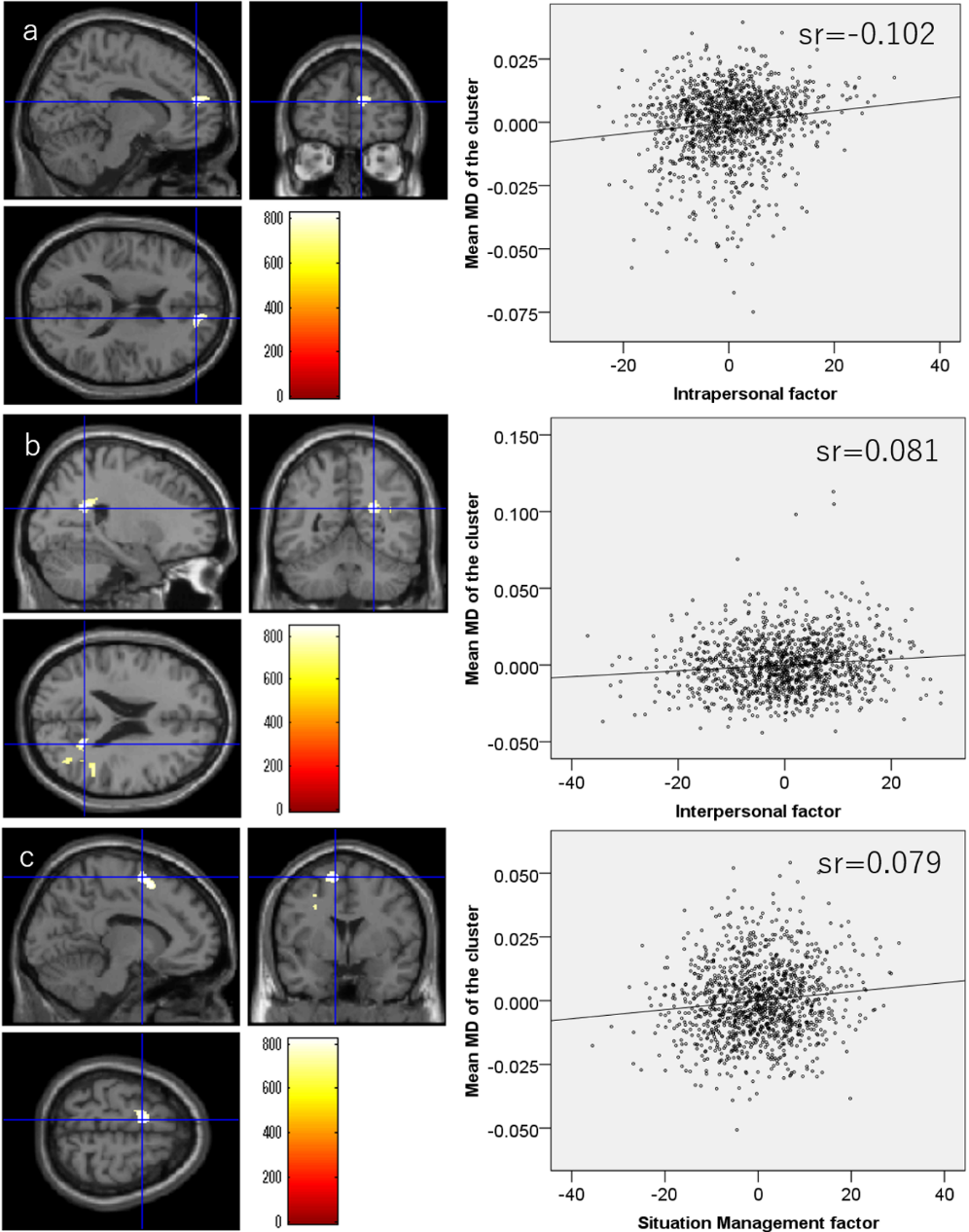


**References**

Bar-On, R. (1997). *Bar-On emotional quotient inventory: Technical manual*. Toronto: Multi-Health Systems.

Barnea-Goraly, N., Menon, V., Eckert, M., Tamm, L., Bammer, R., Karchemskiy, A., Dant, C. C., & Reiss, A. L. (2005). White matter development during childhood and adolescence: a cross-sectional diffusion tensor imaging study. *Cerebral cortex, 15*(12), 1848-1854.

Button, K. S., Ioannidis, J. P., Mokrysz, C., Nosek, B. A., Flint, J., Robinson, E. S., & Munafò, M. R. (2013). Power failure: why small sample size undermines the reliability of neuroscience. *Nature Reviews: Neuroscience, 14*(5), 365-376.

Duzan, H., & Shariff, N. S. B. M. (2015). Ridge regression for solving the multicollinearity problem: review of methods and models. *Journal of Applied Sciences, 15*(3), 392-404.

Fukunishi, I., Wise, T. N., Sheridan, M., Shimai, S., Otake, K., Utsuki, N., & Uchiyama, K. (2001). Association of emotional intelligence with alexithymic characteristics. *Psychological reports, 89*(3), 651-658.

Kanwisher, N., & Yovel, G. (2006). The fusiform face area: a cortical region specialized for the perception of faces. *Philosophical Transactions of the Royal Society B: Biological Sciences, 361*(1476), 2109-2128.

Le Bihan, D., Mangin, J. F., Poupon, C., Clark, C. A., Pappata, S., Molko, N., & Chabriat, H. (2001). Diffusion tensor imaging: concepts and applications. *Journal of Magnetic Resonance Imaging, 13*(4), 534-546.

Magistro, D., Takeuchi, H., Nejad, K. K., Taki, Y., Sekiguchi, A., Nouchi, R., Kotozaki, Y., Nakagawa, S., Miyauchi, C. M., Iizuka, K., Yokoyama, R., Shinada, T., Yamamoto, Y., Hanawa, S., Araki, T., Hashizume, H., Sassa, Y., & Kawashima, R. (2015). The Relationship between Processing Speed and Regional White Matter Volume in Healthy Young People. *PLoS ONE, 10*(9), e0136386.

Maldjian, J. A., Laurienti, P. J., & Burdette, J. H. (2004). Precentral gyrus discrepancy in electronic versions of the Talairach atlas. *Neuroimage, 21*(1), 450-455.

Maldjian, J. A., Laurienti, P. J., Kraft, R. A., & Burdette, J. H. (2003). An automated method for neuroanatomic and cytoarchitectonic atlas-based interrogation of fMRI data sets. *Neuroimage, 19*(3), 1233-1239.

Murphy, S., Norbury, R., Godlewska, B., Cowen, P., Mannie, Z., Harmer, C., & Munafo, M. (2012). The effect of the serotonin transporter polymorphism (5-HTTLPR) on amygdala function: a meta-analysis. *Molecular psychiatry, 18*(4), 512-520.

Oldfield, R. C. (1971). The assessment and analysis of handedness: the Edinburgh inventory. *Neuropsychologia, 9*(1), 97-113.

Open-Science-Collaboration. (2015). Estimating the reproducibility of psychological science. *Science, 349*(6251), aac4716.

Otake, K., Shimai, S., Uchiyama, K., & Utsuki, N. (2001). Development of Japanese Emotional Intelligence Scale (EQS) and its validity and reliability. *Job Stress Research, 8*(3).

Raven, J. (1998). *Manual for Raven's progressive matrices and vocabulary scales*. Oxford: Oxford Psychologists Press.

Salovey, P., & Mayer, J. D. (1990). Emotional intelligence *Imagination, Cognition and Personality, 9*, 185-211.

Salovey, P., Rothman, A. J., Detweiler, J. B., & Steward, W. T. (2000). Emotional states and physical health. *American Psychologist, 55*(1), 110-121.

Schilling, C., Kühn, S., Paus, T., Romanowski, A., Banaschewski, T., Barbot, A., Barker, G., Brühl, R., Büchel, C., & Conrod, P. (2012). Cortical thickness of superior frontal cortex predicts impulsiveness and perceptual reasoning in adolescence. *Molecular Psychiatry, 18*(5), 624-630.

Takeuchi, H., Sekiguchi, A., Taki, Y., Yokoyama, S., Yomogida, Y., Komuro, N., Yamanouchi, T., Suzuki, S., & Kawashima, R. (2010a). Training of Working Memory Impacts Structural Connectivity. *Journal of Neuroscience, 30*(9), 3297-3303.

Takeuchi, H., Taki, Y., Hashizume, H., Asano, K., Asano, M., Sassa, Y., Yokota, S., Kotozaki, Y., Nouchi, R., & Kawashima, R. (2016). Impact of videogame play on the brain’s microstructural properties: Cross-sectional and longitudinal analyses. *Molecular Psychiatry, 21*, 1781-1789.

Takeuchi, H., Taki, Y., Nouchi, R., Sekiguchi, A., Hashizume, H., Sassa, Y., Kotozaki, Y., Miyauchi, C. M., Yokoyama, R., & Iizuka, K. (2013a). Resting state functional connectivity associated with trait emotional intelligence. *Neuroimage, 83*, 318-328.

Takeuchi, H., Taki, Y., Nouchi, R., Sekiguchi, A., Hashizume, H., Sassa, Y., Kotozaki, Y., Miyauchi, C. M., Yokoyama, R., Iizuka, K., Seishu, N., Tomomi, N., Kunitoki, K., & Kawashima, R. (2015a). Degree centrality and fractional amplitude of low-frequency oscillations associated with Stroop interference. *Neuroimage, 119*(1), 197-209.

Takeuchi, H., Taki, Y., Nouchi, R., Yokoyama, R., Kotozaki, Y., Nakagawa, S., Sekiguchi, A., Iizuka, K., Hanawa, S., Araki, T., Miyauchi, C. M., Sakaki, K., Sassa, Y., Nozawa, T., Ikeda, S., Yokota, S., Daniele, M., & Kawashima, R. (2018). General intelligence is associated with working memory-related brain activity: new evidence from a large sample study. *Brain structure & function, Epub ahead of print*.

Takeuchi, H., Taki, Y., Nouchi, R., Yokoyama, R., Kotozaki, Y., Nakagawa, S., Sekiguchi, A., Iizuka, K., Yamamoto, Y., Hanawa, S., Araki, T., Miyauchi, M., Calros , Shinada, T., Sakaki, K., Sassa, Y., Nozawa, T., Ikeda, S., Yokota, S., Daniele, M., & Kawashima, R. (2017). Global associations between regional gray matter volume and diverse complex cognitive functions: evidence from a large sample study. *Scientific Reports, 7*, article 10014.

Takeuchi, H., Taki, Y., Sassa, Y., Hashizume, H., Sekiguchi, A., Fukushima, A., & Kawashima, R. (2010b). Regional gray matter volume of dopaminergic system associate with creativity: Evidence from voxel-based morphometry *Neuroimage, 51*(2), 578-585.

Takeuchi, H., Taki, Y., Sassa, Y., Hashizume, H., Sekiguchi, A., Fukushima, A., & Kawashima, R. (2010c). White matter structures associated with creativity: Evidence from diffusion tensor imaging. *Neuroimage, 51*(1), 11-18.

Takeuchi, H., Taki, Y., Sassa, Y., Hashizume, H., Sekiguchi, A., Fukushima, A., & Kawashima, R. (2011a). Regional gray matter density associated with emotional intelligence: Evidence from voxel-based morphometry. *Human Brain Mapping, 32*(9), 1497-1510.

Takeuchi, H., Taki, Y., Sassa, Y., Hashizume, H., Sekiguchi, A., Fukushima, A., & Kawashima, R. (2011b). Verbal working memory performance correlates with regional white matter structures in the fronto-parietal regions. *Neuropsychologia, 49*(12), 3466-3473

Takeuchi, H., Taki, Y., Sassa, Y., Hashizume, H., Sekiguchi, A., Nagase, T., Nouchi, R., Fukushima, A., & Kawashima, R. (2013b). White matter structures associated with emotional intelligence: Evidence from diffusion tensor imaging. *Human brain mapping, 34*(5), 1025-1034.

Takeuchi, H., Taki, Y., Sekuguchi, A., Hashizume, H., Nouchi, R., Sassa, Y., Kotozaki, Y., Miyauchi, C. M., Yokoyama, R., Iizuka, K., Nakagawa, S., Nagase, T., Kunitoki, K., & Kawashima, R. (2015b). Mean diffusivity of globus pallidus associated with verbal creativity measured by divergent thinking and creativity-related temperaments in young healthy adults. *Human Brain Mapping, 36*(5), 1808-1827.

Takeuchi, H., Taki, Y., Thyreau, B., Sassa, Y., Hashizume, H., Sekiguchi, A., Nagase, T., Nouchi, R., Fukushima, A., & Kawashima, R. (2013c). White matter structures associated with empathizing and systemizing in young adults. *Neuroimage, 77*(15), 222-236.

Taki, Y., Thyreau, B., Hashizume, H., Sassa, Y., Takeuchi, H., Wu, K., Kotozaki, Y., Nouchi, R., Asano, M., & Asano, K. (2013). Linear and curvilinear correlations of brain white matter volume, fractional anisotropy, and mean diffusivity with age using voxel-based and region of interest analyses in 246 healthy children. *Human Brain Mapping, 34*(8), 1842-1856.

Tzourio-Mazoyer, N., Landeau, B., Papathanassiou, D., Crivello, F., Etard, O., Delcroix, N., Mazoyer, B., & Joliot, M. (2002). Automated anatomical labeling of activations in SPM using a macroscopic anatomical parcellation of the MNI MRI single-subject brain. *Neuroimage, 15*(1), 273-289.

Uchiyama, K., Shimai, T., Utsuki, N., & Otake, K. (2001). *EQS manual*. Tokyo: Jitsumukyoiku Syuppan (Practical Education Press).

Vul, E., Harris, C., Winkielman, P., & Pashler, H. (2009). Reply to comments on “puzzlingly high correlations in fMRI studies of emotion, personality, and social cognition”. *Perspectives on Psychological Science, 4*(3), 319-324.

Wechsler, D. (1997). WAIS-III administration and scoring manual. *San Antonio, TX: The Psychological Corporation*.
